# Evaluation of adaptation of the polylactic acid pattern of maxillary complete dentures fabricated by fused deposition modelling technology: A pilot study

**DOI:** 10.1371/journal.pone.0201777

**Published:** 2018-08-29

**Authors:** Kehui Deng, Hu Chen, Yijiao Zhao, Yongsheng Zhou, Yong Wang, Yuchun Sun

**Affiliations:** 1 Center of Digital Dentistry, Peking University School and Hospital of Stomatology, Beijing, China; 2 Department of Prosthodontics, Peking University School and Hospital of Stomatology, Beijing, China; 3 National Engineering Laboratory for Digital and Material Technology of Stomatology, Beijing, China; 4 Research Center of Engineering and Technology for Digital Dentistry of Ministry of Health, Beijing, China; 5 Beijing Key Laboratory of Digital Stomatology, Beijing, China; Istituto Italiano di Tecnologia Center for Micro BioRobotics, ITALY

## Abstract

**Objective:**

To quantitatively evaluate maxillary complete dentures fabricated from polylactic acid (PLA) using fused deposition modelling (FDM) technology.

**Methods:**

A digital maxillary complete denture was prepared based on a standard maxillary edentulous plaster model. The PLA pattern was printed by a FDM machine, with 5 repetitions, while another 5 wax patterns were printed as control group, using a high accuracy three-dimensional (3D) wax printer. The patterns were scanned with a 3D scanner. A light-body silicone film was made after each denture pattern had been totally seated on the plaster model, and was scanned to determine its thickness, which reflected the 3D space between the plaster model and the tissue surface of the denture pattern. The overall area was separated into four parts: primary stress-bearing area, secondary stress-bearing area, border seal area and relief area, and the average deviation of these four parts were measured. The values were analyzed by independent t-test.

**Results:**

The overall mean value and standard deviation of space between PLA denture patterns and plaster model was 0.277 ± 0.021 mm, while that of the wax denture patterns was 0.279 ± 0.045 mm, which showed a good fit overall. No statistically significant (𝑃 > 0.05) difference was observed between the PLA patterns and wax patterns.

**Conclusions:**

The adaptation of the PLA pattern of maxillary complete denture printed by FDM technology is comparable to that prepared by wax printer, and can satisfy the accuracy requirements.

## Introduction

Over the past 30 years, digital technology has come to be widely used in the field of oral medicine. Complete dentures have always posed some problems in traditional prosthodontic treatment, and computer-aided technology is an emerging method for fabricating complete dentures. At present, most digital systems for complete denture production adopt a method of milling a base into which commercial teeth are inserted. [1–3] However, this method increases the likelihood of assembly errors, because each artificial tooth must be inserted into the rigid denture base.

Three-dimensional (3D) printing technology, also known as additive manufacturing technology, which became available in the 1980s, uses a method of layer-by-layer stacking to combine materials to a physical object based on a 3D digital model. In recent years, 3D printing technology has increasingly been applied in the field of prosthodontics, dental implants, orthodontics, surgery guidance, etc. [4, 5] Compared with the traditional subtraction process, 3D printing has inherent advantages, including coping with complex morphology and internal structures, and allowing mixing of various materials during processing. To date, in the dental application of 3D printing, only the use of metal powder and resin materials has been reported, [6, 7] while little is known about the dental application of polylactic acid (PLA) materials with fused deposition modelling (FDM) technology; there are no previous reports of using PLA material for printing a denture pattern.

FDM technology uses high temperature to heat and melt various types of material for deposition modelling. This technology appeared in the late 1980s, and is now widely used for building models in the medical, aerospace, and light industries, etc. [8, 9] PLA is environmentally friendly, comprising biodegradable material (starch raw material extracted from renewable plant resources such as corn), and is widely used in the biomedical field. In addition, it also has good mechanical, elastic modulus, and thermoforming properties, and shows almost no deformation when printing due to its low shrinkage rate. [10–12] Furthermore, the PLA material and FDM machine are relatively cheap, which can reduce the cost of fabricating complete dentures after digital design. A printed PLA custom tray for an edentulous jaw has demonstrated a high reproducibility and accuracy, [13] but higher accuracy is required for a denture pattern; thus, the accuracy of this approach needs to be evaluated further, and is key to its future applications.

This study aimed to evaluate the adaptation of the PLA pattern of maxillary complete dentures printed with an FDM machine, in a quantitative manner, to lay the basis for further clinical applications.

## Materials and methods

### Complete denture CAD

**Fig 1:**
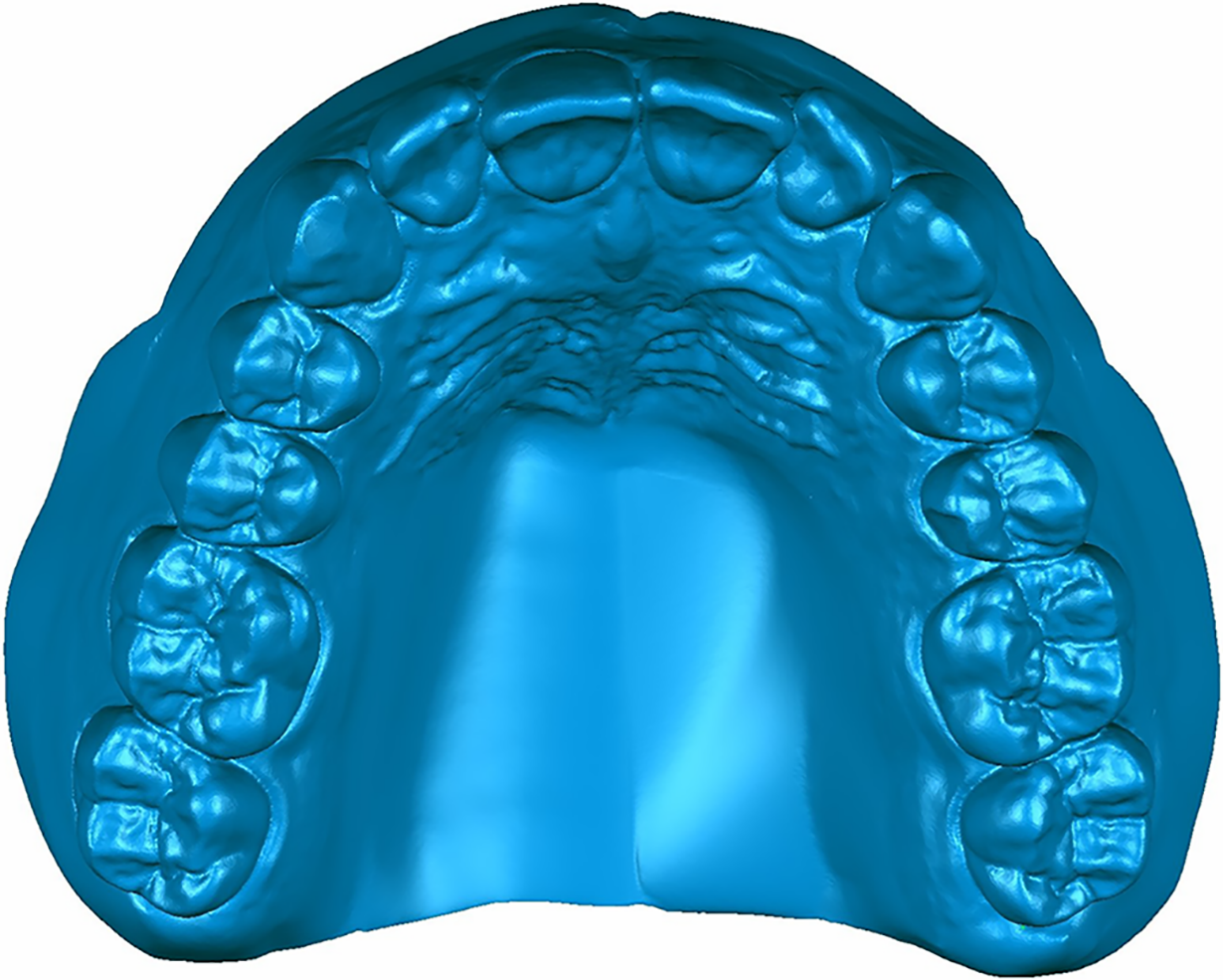
CAD data of complete denture.

The 3D data of the standard edentulous maxillary plaster model and dentition plaster model were obtained using a 3D scanner (Activity 880; SmartOptics, Bochum, Germany) and saved in stereolithography (STL) format. These data were imported into reverse engineering software (Studio 2013, Raindrop Geomagic, Research Triangle, NC, USA). The dentition part of the dental model and the corresponding parts in contact with the denture base of the edentulous model were cut and merged into a complete denture by filling the space between them with triangular mesh data in order to form the thickness of the complete denture. In this way, the complete denture design was formed (Fig 1) and saved in STL file format.

### Denture pattern 3D printing

**Fig 2:**
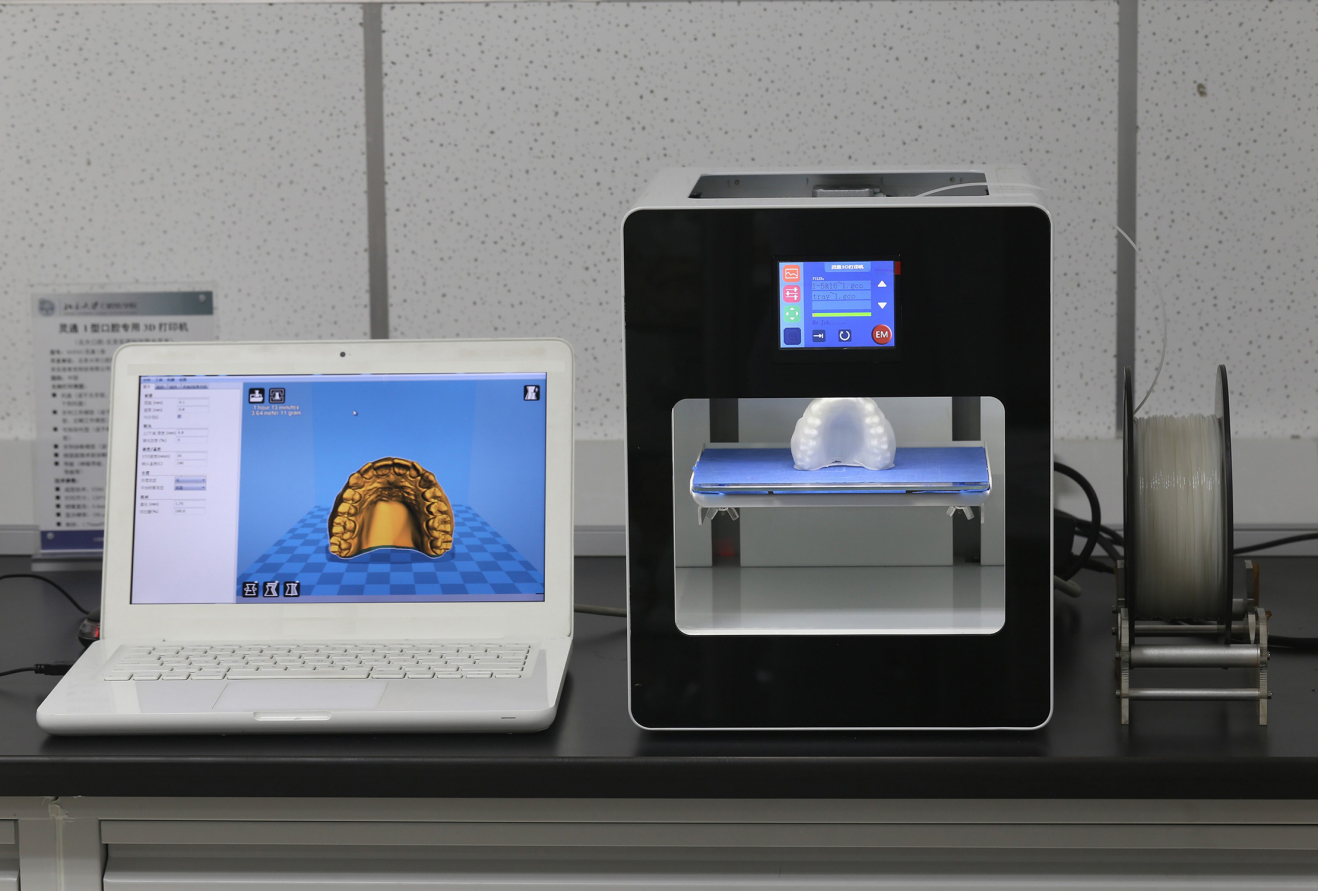
FDM machine and the angle of printing.

**Fig 3:**
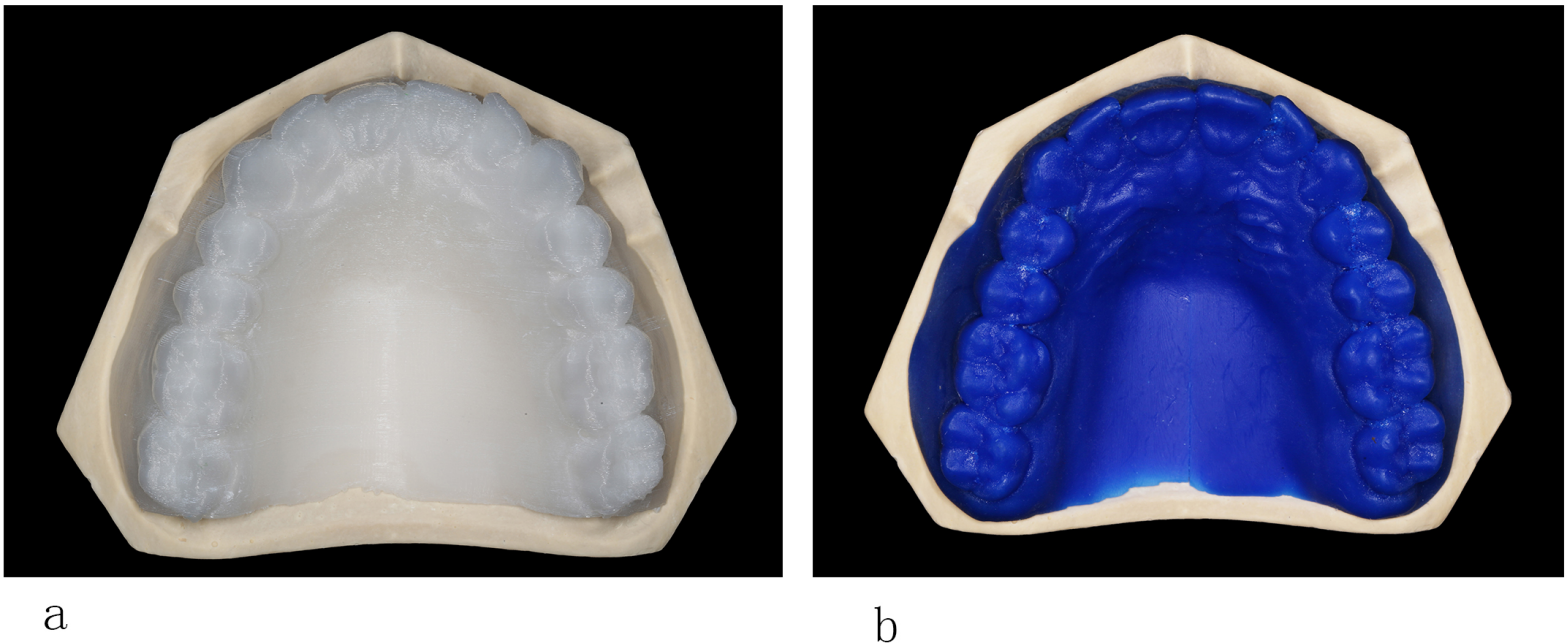
**(a)** PLA pattern of complete denture; **(b)** wax pattern of complete denture.

The complete denture dataset was imported into the supporting software of the FDM 3D printer (Lingtong-II, BeijingShino, Beijing, China). Then, after adjusting the printing angle (Fig 2) and setting the printing mode (0.8-mm diameter nozzle and a layered precision of 0.1 mm, unsupported and 0% infill percentage, with a wall thickness of 0.8 mm), the PLA material of the maxillary complete denture pattern could be printed after the data were section-processed. Additionally, another 5 wax pattern of the same data were printed using a 3D wax printer (ProJet CPX 3500, 3D Systems, Rock Hill, SC, USA) (Fig 3). Scan powder (ECO CHECK ED-ST; MARKTEC, Shanghai, China) was applied to two types of denture pattern surfaces due to reflection of the materials before scanning. After scanning, STL data were out-put (2 groups × 5).

### Preparation of silicone space film

The tissue surfaces of the denture patterns were filled with light-body silicone rubber (Virtual Light Body Regular Set, Ivoclar Vivadent, Liechtenstein) and each pattern was completely seated onto the plaster model; then a load of 5 kg was applied to the pattern for at least 5 min. The operation was completed in a temperature (20°C)-controlled room and the process from injecting the silicon rubber to placing the pattern onto the plaster model was controlled to be within 1 minute. After complete polymerization, the space between the denture pattern and plaster model was filled with silicone. The pattern was removed and the silicone film could be seen attached to the plaster model. The plaster model covered with the silicone film was scanned using the same optical scanner and the data were exported as an STL file (2 groups × 5).

### 3D deviation analysis

The data above were imported into Geomagic software. To analyze the printing error, the scanned data of the printed patterns were registered to the corresponding CAD data, using the multipoint registration command. After registration, a 3D deviation analysis command in Geomagic software was used to measure the deviation between the two data mentioned above (scanned patterns and their CAD data) with the CAD data as the reference and the scanned data as test. The deviation was calculated using average value and Root Mean Square value (RMS). The average value represent the overall error of printing deviation, but this error is the result of positive and negative counteraction, so the RMS (the square root of the arithmetic mean of the squares of the values) is used to eliminate the influence of positive and negative signs when it squared the error, which can better reflect the discreteness of the experimental result errors.

$$RMS=\sqrt{\frac{\sum_{i=1}^{n}x_{i}^{2}}{n}}$$ (1)

**Fig 4:**
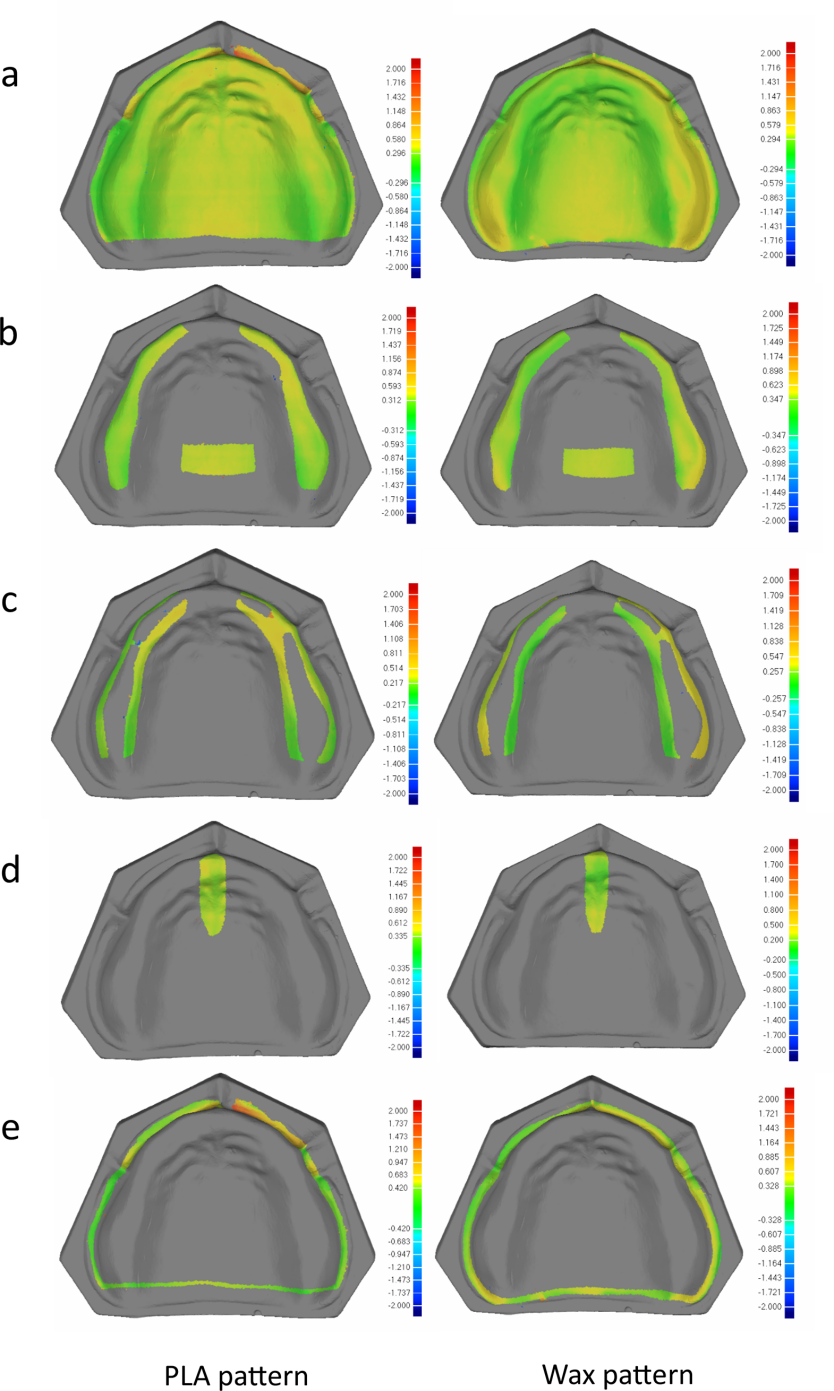
3D deviation analysis of the thickness of the silicone film in Geomagic, green areas show good matching, red indicates a positive error, blue a negative error. **(a)** overall; (**b)** primary stress-bearing area; (**c)** secondary stress-bearing area; (**d)** relief area; (**e)** border seal area.

For quantitative evaluation of the adaptation of the denture pattern, the data of plaster model (without any silicon film) was registered to the data of plaster model with the silicon film using the best-fit alignment command, where the axial plane of the plaster model, that is, the area not covered by silicone film was set as the common area for data registration. So that the thickness of the silicone film was measured by 3D deviation of the surfaces inside the denture marginal line of the data of plaster model with and without silicon film, which represents the space between the plaster model and tissue surface of the denture pattern. The measurements were separated into 4 distinct areas: primary stress-bearing area, secondary stress-bearing area, border seal area and relief area, and the average deviations were recorded to represent the internal adaptation of the corresponding area. Color surface maps were created to generate a visual display of the adaptation of the denture base with the cast overall and in 4 distinct areas. (Fig 4A–4E).

### Statistical analyses

The space between the 2 digitally produced denture patterns was compared using an independent sample t-test. P values equal to or less than 0.05 were considered to be statistically significant. All statistical tests were performed in IBM SPSS Statistics 19 software (IBM SPSS Inc., Chicago, IL, USA)

## Results

As shown in Table 1, overall, the PLA patterns were enlarged compared with the CAD data (average value: 0.016 ± 0.007 mm, RMS: 0.143±0.01 mm); while the wax patterns were smaller than the CAD data (average value: -0.017 ± 0.011 mm, RMS: 0.126±0.007 mm).

**Table 1:** Error in the two printing methods: Deviation between the pattern of complete dentures and CAD data.

Number	Deviation (mm)			
	PLA pattern group		Wax pattern group	
	mean value	RMS	mean value	RMS
1	0.010	0.129	-0.031	0.134
2	0.018	0.141	-0.015	0.12
3	0.020	0.157	-0.026	0.12
4	0.009	0.141	-0.010	0.124
5	0.021	0.146	-0.005	0.132
Mean	0.016 ± 0.007	0.143±0.01	-0.017 ± 0.011	0.126±0.007

As shown in Table 2, the measurement indicates the average space between the tissue surface of the denture pattern and the plaster model, as determined using the silicone film method on 5 denture patterns in each group. The mean value and standard deviation of the space for the PLA patterns was 0.277 ± 0.021 mm, while that of the wax patterns was 0.279 ± 0.045 mm. For all the patterns, the values of the secondary stress-bearing area and the relief area were smaller than that of the primary stress-bearing area and the border seal area. There was no statistically significant difference between PLA pattern and wax pattern by independent *t*-test (P > 0.05).

**Table 2:** Average deviation between the tissue surface of the denture pattern and the plaster model.

Area	Deviation (mm)		P value
	PLA pattern group	Wax pattern group	
Overall	0.277 ± 0.021	0.279 ± 0.045	0.930
Primary stress-bearing area	0.296 ± 0.035	0.344 ± 0.061	0.168
Secondary stress-bearing area	0.213 ± 0.021	0.247 ± 0.064	0.294
Relief area	0.269 ± 0.061	0.257 ± 0.065	0.785
Border seal area	0.338 ± 0.025	0.291 ± 0.053	0.114

## Discussion

**Fig 5:**
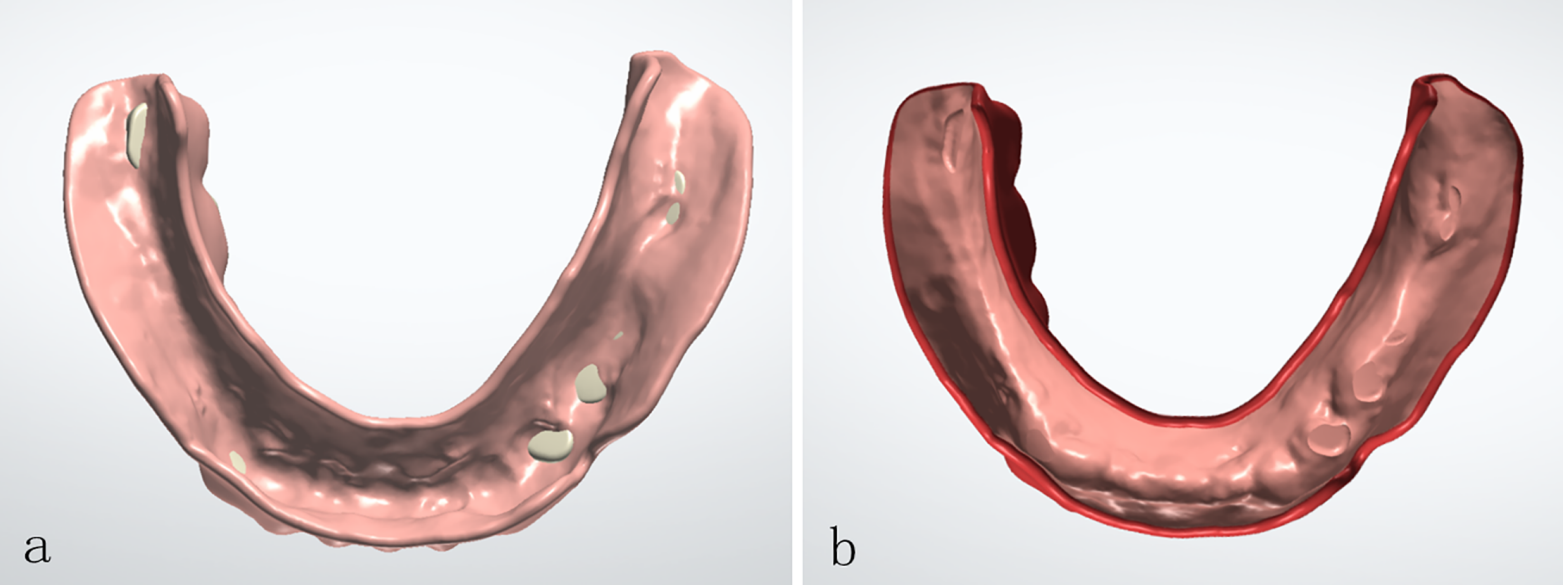
(a) the tissue surface view of complete denture with insufficient prosthetic space (b) improper individual sockets in the base for the teeth are generated (3Shape Dental System 2017, 3Shape, Denmark).

In recent years, many digital complete denture systems have been devised, in which the most common method for fabricating complete dentures is to mill a resin base with individual sockets for each tooth and then manually bond commercial teeth to the milled sockets of the denture. [1] There are two major problems with this segmented manufacturing method. When the commercial teeth are bonded onto the recesses of milled denture base, an undercut in the gingival area could result in gaps between some teeth and the corresponding bases, which can affect the accuracy of tooth position in the denture. [14] Moreover, lack of polymer crosslinking incorporated in commercial teeth may also have reduced strength and hardness. Furthermore, when designing the dentures of a patient with short vertical dimension, insufficient prosthetic space is available for the denture base and the commercial teeth, so that the basal surface of the teeth will pierce the crest of the base, and thus individual sockets in the base for the teeth cannot be generated well in the design software (Fig 5).

Integrated manufacture of complete dentures can effectively avoid the above problems. Integrated multicolor printed dentures are the ideal, but to date, the strength, aesthetics, abrasion resistance, and biosafety of the print processes and materials cannot meet the clinical demands of complete dentures. Therefore, the PLA pattern is printed as an indirect production of complete dentures in this study. The pattern was used to fabricate complete dentures and can also be used for clinical try-in before fabrication. When it is used to manufacture complete dentures, the pattern needs to be fully seated on the plaster model, combined with conventional technology, such as pack and press or injection molding, to form the matrix of the denture. Then the pattern is removed; the commercial teeth (as selected during the design process) are inserted and fixed in the matrix to replace the teeth in the pattern. Special attention is required when using pack and press technology, as the pattern needs to be softened or melted prior to removing it, due to the undercut of the plaster model. PLA material can be soften in water at 100° C allowing PLA patterns to be used with pack and press technology. Finally, the base resin materials are used to fill in matrix to form the base of the denture. However, adaptation of the tissue surface, the accuracy of the polishing surface morphology, and artificial tooth positioning should be evaluated.

Although the polymerization shrinkage of denture base fabricated by the traditional techniques can not be avoided, while the milled denture has less deformation that is an obviously advantage of CAD/CAM technique, the shrinkage of traditional denture base is very low due to the continuous improvement of the base resin material. Studies [15] had shown that though a slight deformation in the border sealing area of traditional denture base compared with the CAD/CAM fabrication, the deformation is still acceptance in the clinic. Except the denture base, the position of teeth is another important part of complete denture, which is directly affect the occlusion grinding time in clinic. However, as mentioned above, the most problem of milled denture is that the rigid bonding between the teeth and milled base could bring more errors in the position of teeth. As a result, the printed patterns in this experiment were used to eliminate this error due to its integrated manufacture, and also, the casting metal network and baseplate can be added in the fabrication of complete, which is impossible for the milled denture. Besides, it has better combining of teeth and base when it combines with the conventional process (polymer crosslinking) and it is not affected by the length of the teeth, and even if the teeth pierce the denture base, it can still be manufactured into a complete denture because the technician can mill the basal part of tooth before inserting it into the matrix. Furthermore, this approach can reduce the cost for manufacturing complete dentures digitally.

The most important issue is whether the pattern can be accurately seated on the plaster model, because inaccurate seating could lead to a series of errors in jaw position and occlusion problems. So, this experiment was designed to evaluate the printing accuracy and adaptation of PLA pattern. In this study, we printed the same data five times to verify the stability and repeatability of the technology, and found that the average standard deviation and RMS of the PLA pattern (in comparison with its CAD data) was 0.016 mm and 0.143mm, which was comparable with the precision of the wax pattern printed using expensive printing equipment. This provided evidence of the high stability and repeatability of FDM printing, and supporting its routine use in clinical practice. The average standard deviation of PLA patterns was positive, indicating slight swelling, while that of wax patterns was negative, indicating slight shrinkage. These errors may be related to the calibration parameters of the printer, emphasizing the need to calibrate the printer regularly.

The adaptation of denture patterns directly determines the effect of the final denture fabricated in the laboratory; hence, in this study, we quantitatively evaluated the adaptation by measuring the space between the tissue surface of the denture pattern and the plaster model. The average standard deviation (Table 2) showed no statistically significant differences between the two groups overall or in 4 sections. The maximum value of PLA group appeared in the border seal area, which may be due to slight swelling, causing poorer fit in the border seal area after the pattern had been totally seated. The maximum value in the wax group was in the primary stress-bearing area, which may be due to slight shrinkage, causing the most convex part in the tissue surface of the pattern (corresponding to the alveolar crest) to be in less contact with the plaster than the rest of the area, due to incomplete seating of the pattern. The minimum value in both groups appeared in the secondary stress-bearing area, which may be because this area is closer to the path of directional placement; thus, the space between the pattern and plaster model does not have a significant effect even with incomplete placement due to slight swelling/shrinkage. The standard deviation of all measured data was within 70 μm, indicating that the pattern printed by these two methods is stable and repeatable, and can be used in clinical applications. However, as all of the test samples were standard models, further clinical evaluation is required.

The accuracy of printed wax patterns of complete dentures has been reported previously. Hu et al. [16] printed a wax pattern of a maxillary complete denture using a 3D wax printer (ProJet CPX 3500, 3D Systems, Rock Hill, SC, USA) with 16-μm forming precision. In their measurement, the space between the tissue surface of the wax pattern and the plaster model was 0.29 ± 0.14 mm, which was consistent with the results of our research. According to the research from Ishinabe Satoshi, [17] after insertion of complete denture, the denture base sinks owing to the effect of bite force and the average deformation of oral mucosa is about 0.3mm, which makes the base and mucosa more fitting, thus ensuring a good effect of border sealing. So, we believe the average of 0.3mm deviation of the space between the tissue surface of pattern and plaster model is acceptable. However, there are many limitations to printing wax pattern of complete dentures. It takes a long time to print a maxillary complete denture pattern (14–16 h) and to remove the support material (1–2 h), and it also requires an expensive wax printer and raw materials, limiting its routine clinical use. In contrast, FDM technology and PLA material can save time and costs. Thus, this is a feasible approach for printing complete denture patterns.

In this study, the infill percentage was set to 0% to reduce the shrinkage of the material and to shorten the printing time, as compared to a 100% infill percentage. The infill percentage is used to change the interior solidity of the 3D print; 0% is completely hollow and 100% is completely solid. Moreover, a zero-fill structural pattern can easily be softened in boiling water and taken out to manufacture the final complete denture. Compared with wax patterns, the time for producing a PLA denture pattern was greatly shortened (about 1 h) and the cost was greatly reduced, and there was less deformation when seating it on the plaster model or during try-in on the alveolar crest. As compared with the traditional manual method, the CAD&FDM protocol can reduce the time-consuming manual laboratory procedures and improve the production efficiency of complete denture patterns.

However, there are still some limitations in the process of printing a PLA pattern using an FDM machine: The angle at which the denture pattern is placed for printing will have a certain impact on the printing accuracy. The unsupported mode was used to achieve surface smoothness of the pattern, but the hanging part, which has no support, will be prone to more errors. In order to reduce the printing errors and ensure the success of the printing progress, the denture was placed vertically, and the postdam area was set downwards during printing (Fig 2); however, this placement angle will produce some error on the palatal side of the denture base in the region of the anterior teeth. There is thus a need for manual adjustment so that the pattern can be fully seated on the plaster model to obtain accurate artificial dentition. Further research is required to establish the best approach for adjusting this angle; and achieving non-defective printing of the key areas.

A 0.8-mm diameter nozzle was used to shorten the printing time to about 1 hour for printing a maxillary complete denture pattern. If higher accuracy is required, for instance to show more details, a smaller diameter nozzle can be used, but the printing time may be extended. However, the printing process that does not require manual intervention, and can therefore save valuable labor hours, thus still offering a technical advantage over the manual approach. In future, final complete dentures based on this technology should be clinically evaluated, establish its usefulness in dental practice.

## Conclusions

In this study, the adaptation of a maxillary complete denture pattern printed by FDM technology, was found to be comparable to that produced using the wax printing method. Used together with CAD software for producing a pattern of complete dentures, it can replace the traditional manual approach and improve the functional suitability of the final complete denture. The PLA pattern printed using an FDM machine can be a low-cost substitute for manufacturing complete dentures and the process satisfies accuracy requirements.

## Supporting information

S1 Dataset Data record of the experiment. This table contains a summarized table and detailed data of printing accuracy and the deviation between the denture pattern and plaster model.
(XLSX)
